# Supplementary material for: Gpr158 Deficiency Impacts Hippocampal CA1 Neuronal Excitability, Dendritic Architecture, and Affects Spatial Learning
Source: Front Cell Neurosci. 2019 Oct 25;13:465. doi: 10.3389/fncel.2019.00465 (PMC6843000; doi:10.3389/fncel.2019.00465)
Supplement: Supplementary file 1 [file Data_Sheet_1.pdf]

Supplemental figures and tables to:

Gpr158 deficiency impacts hippocampal CA1 neuronal excitability, dendritic architecture, and affects spatial learning

Demirhan Çetereisi*[,1], Ioannis Kramvis*[,1], Titia Gebuis[1], Rolinka J. van der Loo[1], Yvonne Gouwenberg[1], Huibert D. Mansvelder[2], Ka Wan Li[1], August B Smit[1], Sabine Spijker[1]

Contains:

Supplemental Figure 1: *Gpr158* KO mice and *Gpr158* gene expression in the hippocampus
Supplemental Figure 2: *Gpr158* KO mice show a MWM acquisition deficit but normal open field and contextual fear memory.
Supplemental Figure 3: *Gpr158* knock-down (KD) in hippocampal culture.
Supplemental Figure 4: Active properties of *Gpr158* KO neurons are independent of membrane potential.
Supplemental Figure 5: Principle component analysis of morphological and electrophysiological parameters.

Supplemental Table 1: Overview statistics behavioral assessments.
Supplemental Table 2: Overview statistics spontaneous EPSCs / IPSCs, and evoked responses.
Supplemental Table 3: Overview statistics *in vitro* morphological analysis.
Supplemental Table 4: Overview statistics *ex vivo* morphological and AP profile analysis.
Supplemental Table 5: Overview statistics of morphological analysis for correlation with behavior.
Supplemental Table 6: Overview of hippocampus subregion gene expression of ECM-related Gpr158 interactors.

Note: Supplemental Table 7, containing the raw data values of figures, is provided separately as excel file.

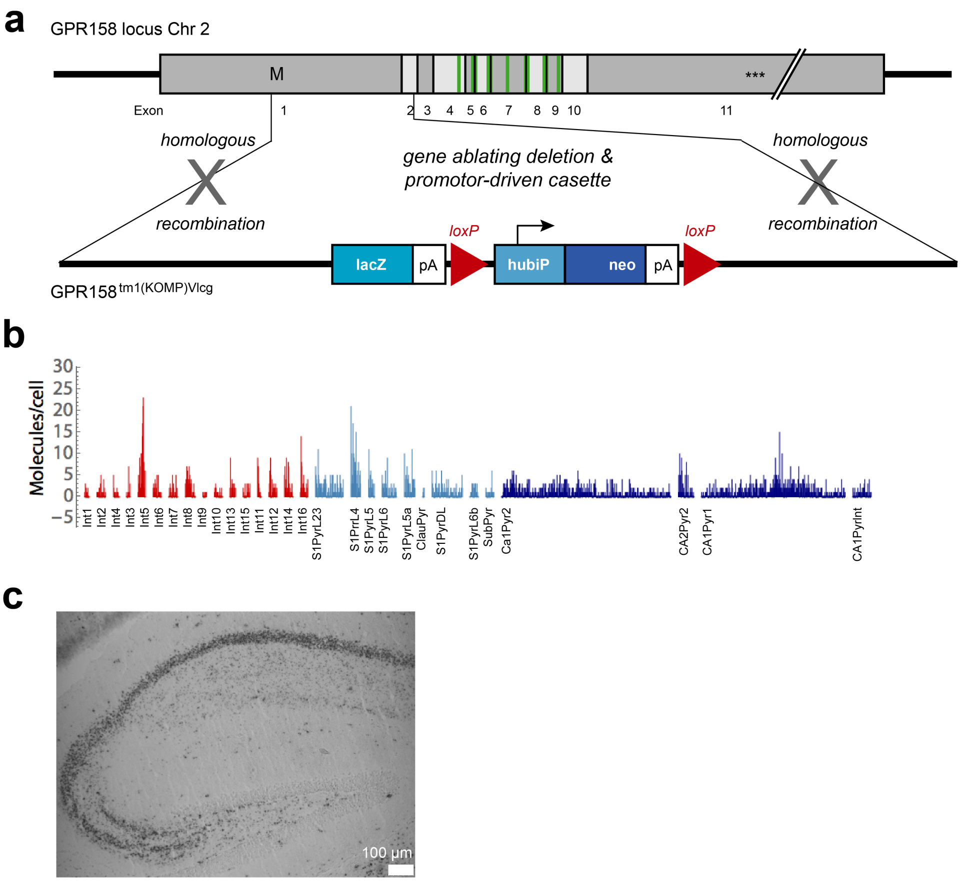

Supplemental Figure 1. ***Gpr158* KO mice and *Gpr158* gene expression in the hippocampus**. **a**) Depicted is the exon structure of Gpr158 with 11 exons, with the approximate start site (M), stop site (***) and transmembrane regions (green bars). Schematic representation of the generation of *Gpr158* KO mice with the *Gpr158* locus and the Gpr158 KOMP construct (VG10108) used for homologous recombination (Gpr158$^{tm1(KOMP)Vlcg}$), generated previously (Orlandi et al., 2015), in which half of exon 1 and exon 2 were replaced by a LacZ cassette. **b**) *Gpr158* transcript levels based on the single cell hippocampal and cortical RNAseq online resource provided by (Huang and Thathiah, 2015; Leung and Wong, 2017). *Gpr158* is expressed in CA1 pyramidal neurons (dark blue), as well as in different interneurons (red) of the cortex and hippocampus. **c**) LacZ staining in *Gpr158* KO mice shows activity of the *Gpr158* promotor in the hippocampus CA1to CA3 region. Scale bar is indicated.

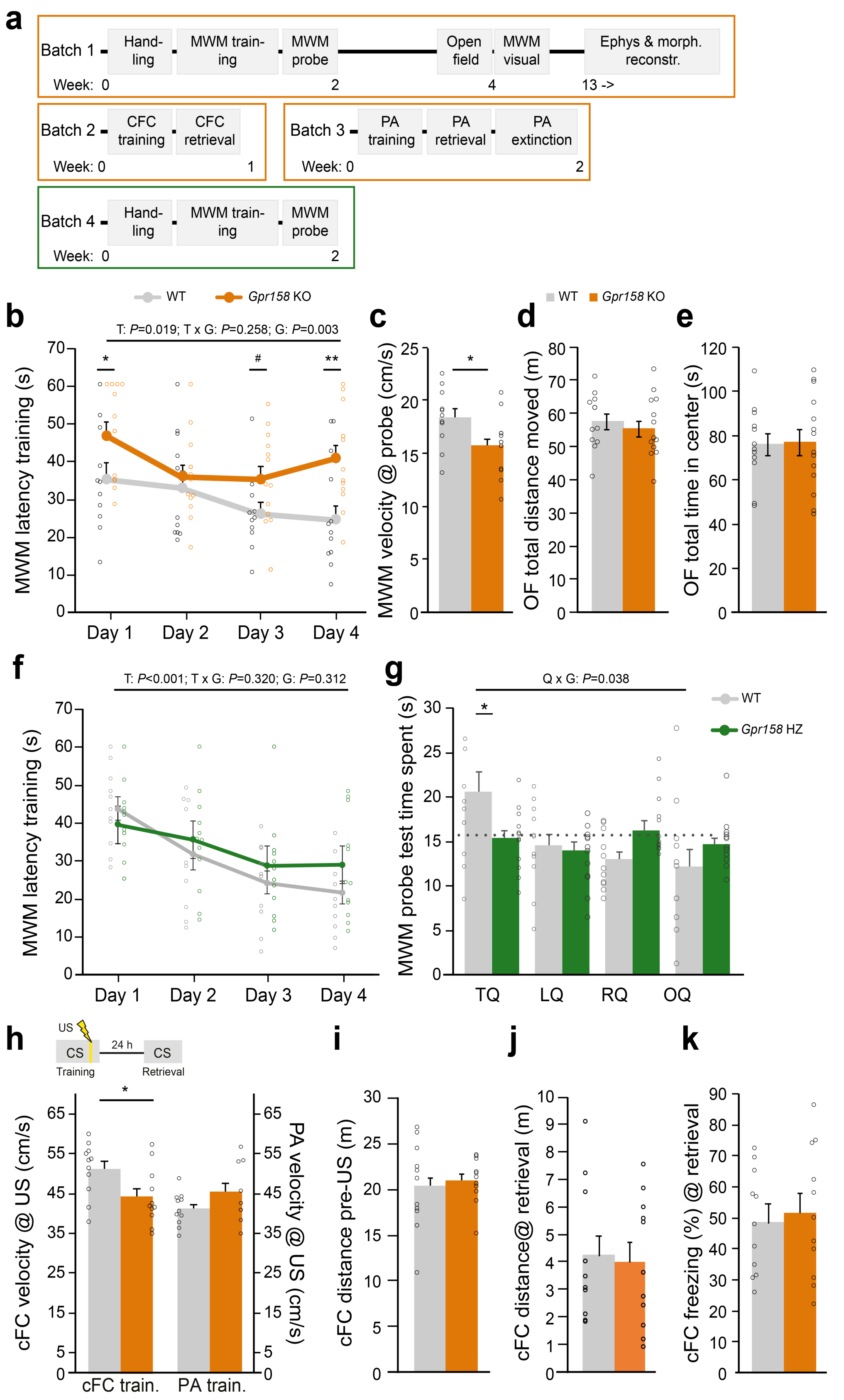

Supplemental Figure 2. ***Gpr158* KO mice show a MWM acquisition deficit but normal open field and contextual fear memory**. **a**) Overview of the 4 batches used for behavioral analysis (orange, *Gpr158* KO; green *Gpr158* HZ). **b,c**) MWM latency to reach the platform during training (c) showed an overall similar effect as distance

(see Fig. 1a) with a genotype (G, *P*=0.003) and training (T, *P*=0.019) effect. *Gpr158* KO mice showed a significant difference in the mean velocity during the probe test (d), arguing that distance to find the platform during acquisition best reflects true genotype differences. **d,e**) Open field (OF) total distance moved (e; *P*=0.509), and total time spent in the center (f; *P*=0.909) showed no genotype differences (*Gpr158* KO n=14, WT n=12). **f,g**) MWM latency to reach the platform during training of HZ (green) *vs.* WT mice (c) showed a normal learning pattern with only a training effect (T, *P*<0.001). Both WT and HZ mice showed learning from day 1 to 4 (WT: *P*<0.001; HZ: *P*=0.029). During the probe test, a significant quadrant x genotype effect was apparent (*P*=0.038), reflecting the inability of *Gpr158* HZ mice to locate the platform (genotype: *P*=0.042). **h-k**) Experimental set-up and data for testing long-term contextual fear memory (cFC). Mice received a foot shock (unconditioned stimulus, US) in the training context (conditioned stimulus, CS). Long-term memory was measured 24 h after training by placing the mouse back in the CS and measuring freezing level. *Gpr158* KO mice showed a small decrease in velocity after delivery of the foot shock in the CFC (h, left axis; *P*=0.031), possibly indicating a difference in shock perception. However, as this was not observed with the same shock intensity in the PA test in an independent batch (h, right axis; *P*=0.172, see Fig 1c), this is most likely a batch effect. *Gpr158* KO mice did not show a difference in terms of distance moved either during training prior to US delivery (i, *P*=0.715), nor 24 h later at memory retrieval (j, *P*=0.829). *Gpr158* KO mice did not show an impaired long-term memory in terms of their freezing level (k; *P*=0.693) upon memory retrieval, corroborating the intact PA memory (see Fig. 1c). Data are presented as mean±SEM with individual data points indicated. Asterisks and octothorpe indicate the level of significance between WT and KO, or WT and HZ assessed by two-tailed Student's t-test or MWU (Supplemental Table 1). # *P*≤0.10; * *P*≤0.050; ** *P*≤0.010.

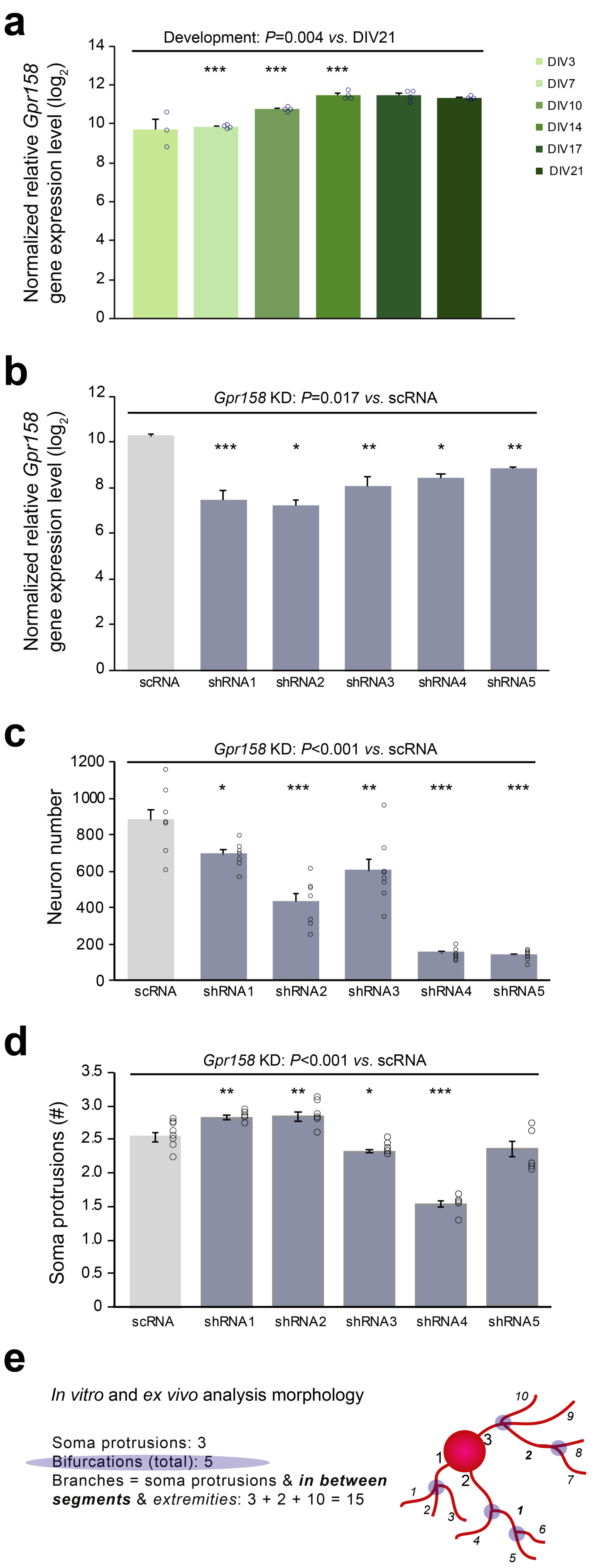

Supplemental Figure 3. ***Gpr158* knock-down (KD) in hippocampal culture**. **a**) Endogenous gene expression level of *Gpr158* from an *in vitro* hippocampal primary culture (n=4 wells, except for DIV3, n=3 wells) during development (DIV3-21). *Gpr158* showed an overall developmental increase in gene expression levels (Kruskal-Wallis, *P*=0.004), with expression being significantly different from DIV7 to DIV14 as compared to DIV21. **b**) Knock-down (KD) efficiency of five shRNAs against the *Gpr158* gene. Neurons were transduced at DIV7 and harvested at DIV14 Overall, all shRNAs significantly reduced *Gpr158* gene expression level compared with the scrambled control (scRNA) (Kruskal-Wallis, *P*=0.017). **c**) *Gpr158* KD effect on neuron number in hippocampal primary cultures. Neurons were transduced at DIV7 and the neuron number was counted at DIV14. It is of note that the large reduction in neuronal number in shRNA4 and 5 might have left a higher proportion of non-transduced neurons alive, resulting in more variable results with respect to endogenous *Gpr158* expression (see panel b), or to specific morphological parameters (see Figure 3, and panel d). **d**) *Gpr158* KD effect on number of neuronal protrusions from the cell soma (see panel e) was highly variable, with shRNA1 and 2 yielding an increase, and shRNA3 and 4 yielding a significant decrease. This could indicate off-target effects, a non-representative measurement parameter, or the fact that specifically in shRNA1 and 2, showing the least overall effects, that the *Gpr158* KD elicited a homeostatic response. **e**) Schematic representation of morphological parameters analyzed for *in vitro* (see Figure 3) and *ex vivo* (see Figure 4). Purple shaded circles indicate the number of bifurcations. Data are presented as mean±SEM with individual data points indicated. Asterisks indicate the level of significance between WT and KO assessed by Student's t-test (Supplemental Table 3). * *P*≤0.050; ** *P*≤0.010; *** *P*≤0.001.

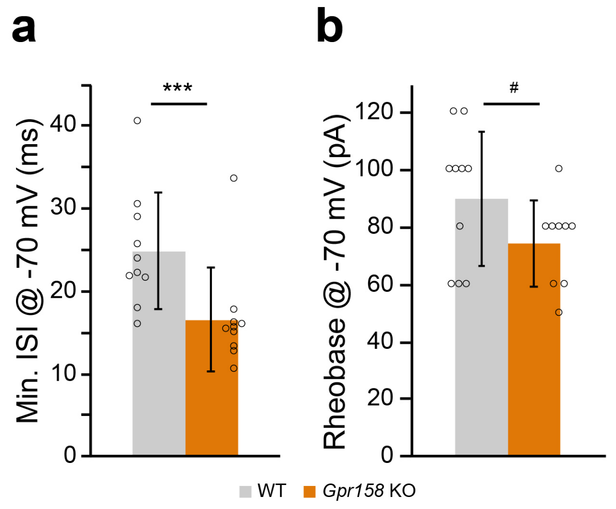

Supplemental Figure 4. **Active properties of *Gpr158* KO neurons are independent of membrane potential. a**) When the membrane potential was clamped at -70 mV, the minimum inter spike interval was significantly reduced in *Gpr158* KO pyramidal cells. **b**) In addition, a trend for lowered rheobase was observed. Data are presented as mean±SD; individual data points are indicated. Asterisks indicate significant differences compared with WT cells assessed by Student's t-test or MWU (Supplemental Table 4), # $P<0.100$; *** $P\leq0.001$.

a

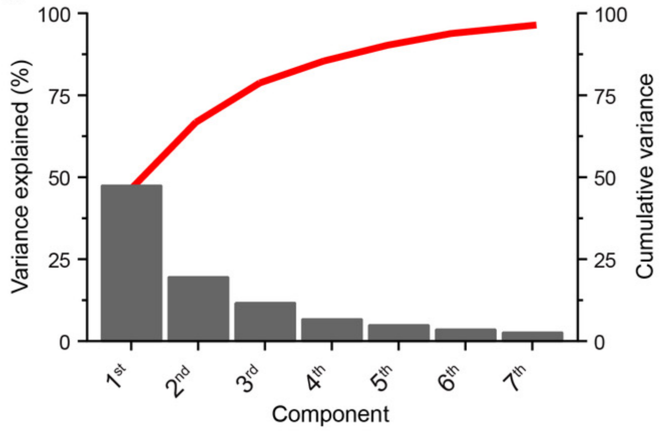

	Variance explained (%)	Cumulative variance
Component 1	47.4815623	47.481562
Component 2	19.5554565	67.037019
Component 3	11.7411817	78.778200
Component 4	6.67756245	85.455763
Component 5	4.85807496	90.313838
Component 6	3.52860925	93.842447
Component 7	2.63547906	96.477926
Component 8	1.84516526	98.323091
Component 9	0.70357794	99.026669
Component 10	0.51644760	99.543117
Component 11	0.21526819	99.758385
Component 12	0.16613684	99.924522
Component 13	0.06017517	99.984697
Component 14	0.01404516	99.998742
Component 15	0.00125764	100

b

Principal Component Analysis

	1st Comp.	2nd Comp.	3rd Comp.	Cell surface area *vs.* Pearson *r*	*P*-value	*P*-value corrected
Length	467.041	115.618	220.894	0.9789	0.0000	**0.0000**
Surface	6213.502	1940.175	2627.977	1.0000	0.0000	**0.0000**
Bifurcations	4.347	1.140	3.214	0.9348	0.0000	**0.0000**
Branches	8.891	2.386	6.747	0.9447	0.0000	**0.0000**
AP Amplitude	-0.001	-3.069	0,989	-0.1324	0.5779	-
AP threshold	0.066	1.374	-0.338	0.1518	0.5229	-
AP min ISI	1.809	1.140	0.428	0.7571	0.0001	**0.0011**
Rheobase free	8.080	-1.496	-3.452	0.6681	0.0013	**0.0115**
Vm	-0.938	1.868	-0.460	-0.2261	0.3379	-
Ri	-11.607	4.737	-4.347	-0.8458	0.0000	**0.0000**
TauM	0.743	0.813	0.878	0.3648	0.1138	-
PPR 1st Raw Amplitude	10.573	-3.618	-16.781	0.5277	0.0168	0.1343
PPR 2nd Raw Amplitude	16.449	-11.538	-22.662	0.4893	0.0286	0.1999
PPR	-0.014	-0.096	0.106	-0.1428	0.5482	-
Stimulation Amplitude @ 200	25.717	-25.706	-59.982	0.3493	0.1312	-

Supplemental Figure 5. **Principle component analysis of morphological and electrophysiological parameters**. **a**) Graph showing the variance explained by the first 7 PCA components (bars) and cumulative (red line). Table with all 15 components deduced from PCA of morphological and electrophysiological parameters measured from the same set of pyramidal neurons (see Figure 4). **b**) The coefficients for the different morphological and electrophysiological parameters of the first 3 components are indicated, demonstrating cell surface area as the most prominent determinant. To the right, the correlation matrix between cell surface area against all variables included in the PCA, including the Pearson correlation *r*, *P*-value, and multiple comparison corrected *P*-value (bold when significant).

Figure reference	n-number	type of overall or post-hoc test	(Df) F- or t-value	*P*-value	Remarks
**1a**: MWM training distance (m)	KO: 13 (& 1 floater taken out)<br>WT: 11 (& 1 floater taken out)	• mixed ANOVA: repeated measure (time) ANOVA (genotype)<br>• ttest	• time: (3,66) 0.80; time x genotype: (3,66) 2.61; genotype: (1,22) 4.49<br>• day 1: (22) -1.29; day 2: (22) 0.53; day 3: (22) -2.33; day 4: (22) -2.41	• time: **<0.001**; time x genotype: **0.008**; genotype: **0.033**<br>• day 1: 0.209; day 2: 0.600; day 3: **0.031**; day 4: **0.025**	Sphericity assumed (ANOVA); day 3: unequal variance
**1b**: MWM probe test time spent (s)	KO: 11<br>WT: 14	• mixed ANOVA: repeated measure (quadrant) ANOVA (genotype)<br>• ttest	• quadrant x genotype: (3,66) 4.46; quadrant for WT: (3,30) 3.45; quadrant for KO: (3,36) 2.23<br>• TQ: (22) 2.88; LQ: (22) 0.88; RQ: (19.5) -1.43; OQ: (22) -2.26	• quadrant x genotype: **0.007**; quadrant for WT: **0.029**; quadrant for KO: 0.102<br>• TQ: **0.009**; LQ: 0.391; RQ 0.168; OQ **0.034**	Sphericity assumed (ANOVA); RQ: unequal variance
**1c**: PA retrieval latency to enter (s)	KO: 9<br>WT: 11	MWU		0.656 (MWU)	non-normal
**1d**: PA extinction latency to enter (s)	KO: 9<br>WT: 11	• mixed ANOVA: repeated measure (time) ANOVA (genotype)<br>• ttest	• time: (2.34,42.20) 20.72; time x genotype: (2.34,42.20) 2.82; genotype: (1,18) 0.025<br>• day 1: (11.45) -2.14; day 2: (10.05) -2.37; day 3: (10.33) -1.97; day 4: (9.69) -1.74	• time: **<0.001**; time x genotype: 0.065; genotype: 0.914<br>• day 1: 0.054; day 2: **0.039**; day 3: 0.077; day 4: 0.114	Huynh-Feldt (ANOVA); day 1-4: unequal variance
**SF2b**: MWM training latency (s)	KO: 13<br>WT: 11	• mixed ANOVA: repeated measure (time) ANOVA (genotype)<br>• ttest or MWU	• time: (3,66) 3.55; time x genotype: (3,66) 1.38; genotype: (1,22) 11.50<br>• day 3: (22) -1.91; day 4 (22) -3.06	• time: **0.019**; time x genotype: 0.258; genotype: **0.003**<br>• day 1: **0.041** (MWU); day 2: 0.494 (MWU); day 3: 0.069 (ttest; day 4: **0.006** (ttest)	Sphericity assumed (ANOVA); day 1 & 2: non-normal
**SF2c**: MWM distance probe test (m)	KO: 13<br>WT: 11	ttest	(22) 2.39	**0.026**	
**SF2d**: OF total distance moved (m)	KO: 14<br>WT: 12	ttest	(24) 0.67	0.509	
**SF2e**: OF time spent in center (s)	KO: 14<br>WT: 12	ttest	(24) -0.12	0.909	
**SF2f**: MWM training latency (s)	HZ: 13<br>WT: 11	• mixed ANOVA: repeated measure (time) ANOVA (genotype)	• time: (3,66) 310.32; time x genotype: (3,66) 1.19; genotype: (1,22) 1.07	time: **<0.001**; time x genotype: 0.320; genotype: 0.312	Sphericity assumed (ANOVA)
**SF2g**: MWM probe test time spent (s)	HZ: 13<br>WT: 11	• mixed ANOVA: repeated measure (quadrant) ANOVA (genotype)<br>• ttest	• quadrant x genotype: (2.45,54.01) 128.18; quadrant for WT: (2.00,20.04) 3.84; quadrant for HZ: (3,36) 0.73<br>• TQ: (13.04) 2.26	• quadrant x genotype: **0.038**; quadrant for WT: **0.039**; quadrant for HZ: 0.539<br>• TQ: **0.042**	Huynh-Feldt (ANOVA); WT, Huynh-Feldt (ANOVA); HZ, Sphericity assumed (ANOVA)<br>TQ: unequal variance
**SF2h**: cFC training, Vmean at US	KO: 11<br>WT: 11	ttest	(20) 2.33	**0.031**	
**SF2i**: PA Vmean at US	KO: 9<br>WT: 11	ttest	(11.79) -1.54	0.172	unequal variance
**SF2j**: cFC training, distance pre-US (m)	KO: 11<br>WT: 11	ttest	(15.09) -0.37	0.715	unequal variance
**SF2k**: cFC training, distance @ retrieval (m)	KO: 11<br>WT: 11	ttest	(20) 0.22	0.829	
**SF2k**: cFC retrieval, % freezing	KO: 11<br>WT: 11	ttest	(20) -0.40	0.693	
Main text: Visual test latency	KO: 13<br>WT: 11	ttest	(18.10) -1.33	0.201	unequal variance

Supplemental Table 1. **Overview statistics behavioral assessments**. Shown are the statistical analyses (n-number, type of test, Df/F/t-value, *P*-value) for the data shown in Figure 1, and Supplemental Figure 2 (SF2) related to behavior in *Gpr158* KO and WT animals. Significance (*P*<0.050) is indicated in bold, trend (*P*<0.100) in underlined.

Figure reference	n-number	type of overall or post-hoc test	(Df) & F- or t-value	*P*-value	Remarks
**2b**: Frequency sEPSCs (Hz)	KO: 14 (4 animals)<br>WT: 10 (4 animals)	ttest	(22) 1.34	0.193	unequal variance
**2b**: Current sEPSCs (pA)	KO: 14 / 4<br>WT: 10 / 4	MWU		0.509	non-normal
**2b**: Decay time sEPSCs (ms)	KO: 14 / 4<br>WT: 10 / 4	ttest	(20.3) -2.12	**0.047**	unequal variance
**2b**: Rise time sEPSCs (ms)	KO: 14 / 4<br>WT: 10 / 4	ttest	(20.3) -1.39	0.179	
**2d**: Frequency sIPSCs (Hz)	KO: 14 / 4<br>WT: 11 / 4	ttest	(23) 0.734	0.470	
**2d**: Current sIPSCs (pA)	KO: 14 / 4<br>WT: 11 / 4	MWU	-	-	non-normal
**2d**: Decay time sIPSCs (ms)	KO: 14 / 4<br>WT: 11 / 4	ttest	(23) -0.01	0.995	
**2d**: Rise time sIPSCs (ms)	KO: 14 / 4<br>WT: 11 / 4	ttest	(23) -1.03	0.314	
**2f**: input-output	KO: 10 / 3<br>WT: 10 / 4	• mixed ANOVA: repeated measure (stimulation) ANOVA (genotype)<br>• MWU	• stimulation: (1.34,24.13) 41.4; time x genotype: (1.34,24.13) 9.36; genotype: (1,18) 8.37	• stimulation: **<0.0001**; stimulation x genotype: **0.003**; genotype: **0.001**<br>• 20 ms: 0.315; 40 ms: **0.023**; 60 ms: **0.003**; 80 ms: **0.003**; 100 ms: **<0.001**; 120 ms: **0.001**; 140 ms: **0.001**; 160 ms: **0.002**; 180 ms: **<0.001**; 200 ms: **0.001**	• Greenhouse-Geisser<br>• All non-normal
**2g**: Paired-pulse ratio	KO: 10 / 3<br>WT: 10 / 4	ttest	(12.8) 0.06	0.954	unequal variance
**2h**: Current (pA) by pulse	KO: 10 / 3<br>WT: 10 / 4	ttest	1st pulse: (18) 5.01<br>2nd pulse: (18) 5.70	1st pulse: **<0.001**<br>2nd pulse: **<0.001**	

Supplemental Table 2. **Overview statistics spontaneous EPSCs / IPSCs, and evoked responses**. Shown are the statistical analyses (n-number (slice / animal), type of test, Df/F/t-value, *P*-value) for the data shown in Figure 2, related to electrophysiological assessment in *Gpr158* KO and WT CA1. Significance (*P*<0.050) is indicated in bold.

Figure reference	n-number	type of overall or post-hoc test	(Df) & F- or t-value	*P*-value	Remarks
**3c**: Total neurite length	Sc: 8 wells<br>shRNA1: 7 wells;<br>shRNA2: 7 wells;<br>shRNA3: 8 wells;<br>shRNA4: 7 wells;<br>shRNA5: 8 wells	• Kruskal-Wallis (all shRNAs *vs.* Sc)<br>• ttest *vs.* Sc	• -<br>• shRNA1: (13) -0.16; shRNA2: (13) 0.83; shRNA3: (14) 7.53; shRNA4: (8.33) 1.73; shRNA5: (14) 8.58	• treatment: <**0.001**<br>• shRNA1: 0.870; shRNA2: 0.424; shRNA3: **<0.001**; shRNA4: 0.120; shRNA5: **<0.001**	• Unequal variance in ANOVA<br>• shRNA4, unequal variance
**3d**: Bifurcation	See above	• Kruskal-Wallis (all shRNAs *vs.* Sc)<br>• ttest *vs.* Sc	• -<br>• shRNA1: (13) 0.735; shRNA2: (13) 2.297; shRNA3: (7.724) 7.600; shRNA4: (7.064) -0.614; shRNA5: (8.040) 8.433	• treatment: **<0.001**<br>• shRNA1: 0.476; shRNA2: **0.039**; shRNA3: **<0.001**; shRNA4: 0.558; shRNA5: **<0.001**	• Unequal variance in ANOVA<br>• shRNA3-5, unequal variance
**3e**: Extremities	See above	• Kruskal-Wallis (all shRNAs *vs.* Sc)<br>• ttest *vs.* Sc	• -<br>• shRNA1: (13) 0.244; shRNA2: (13) 1.393; shRNA3: (14) 7.024; shRNA4: (8.149) 0.793; shRNA5: (14) 7.908	• treatment: **<0.001**<br>• shRNA1: 0.811; shRNA2: 0.187; shRNA3: **<0.001**; shRNA4: 0.450; shRNA5: **<0.001**	• Unequal variance in ANOVA<br>• shRNA4, unequal variance
**SF3a**: Normalized relative *Gpr158* expression level (log2)	DIV3: 2 wells; rest: 4 wells	• Kruskal-Wallis (development)<br>• ttest *vs.* DIV21	• -<br>• DIV3: (1.03) 5.04; DIV7: (6) 20.66; DIV10: (6) 7.03; DIV14: (6) 7.03; DIV17: (6) -0.86	• development: **0.004**<br>• DIV3: 0.119; DIV7: **<0.001**; DIV10: **<0.001**; DIV14: **<0.001**; DIV17: 0.421	• Unequal variance in ANOVA<br>• DIV3: unequal variance
**SF3b**: Normalized relative *Gpr158* expression level (log2)	shRNA5: 2 wells; rest: 3 wells	• Kruskal-Wallis (all shRNAs vs. Sc)<br>• ttest *vs.* Sc	• -<br>• shRNA1: (4) 19.44; shRNA2: (2.13) 6.47; shRNA3: (4) 9.08; shRNA4: (2.15) 4.12; shRNA5: (4) 8.15	• treatment: **0.017**<br>• shRNA1: **<0.001**; shRNA2: **0.020**; shRNA3: **0.001**; shRNA4: **0.048**; shRNA5: **0.004**	• Unequal variance in ANOVA<br>• shRNA2 & shRNA4: unequal variance
**SF3c**: Neuron number	Sc: 8 wells<br>shRNA1: 7 wells;<br>shRNA2: 7 wells;<br>shRNA3: 8 wells;<br>shRNA4: 7 wells;<br>shRNA5: 8 wells	• Kruskal-Wallis (all shRNAs *vs.* Sc)<br>• ttest vs. Sc	• -<br>• shRNA1: (13) 2.70; shRNA2: (13) 5.61; shRNA3: (14) 3.15; shRNA4: (7.54) 11.74; shRNA5: (7.36) 11.95	• treatment: **<0.001**<br>• shRNA1: **0.018**; shRNA2: **<0.001**; shRNA3: **0.007**; shRNA4: **<0.001**; shRNA5: **<0.001**	• Unequal variance in ANOVA<br>• shRNA4&5, unequal variance
**SF3d**: Soma protrusions	See above	• Kruskal-Wallis (all shRNAs *vs.* Sc)<br>• ttest *vs.* Sc	• -<br>• shRNA1: (8.75) -3.82; shRNA2: (13) -3.04; shRNA3: (14) 2.84; shRNA4: (13) 11.60; shRNA5: -	• treatment: **<0.001**<br>• shRNA1: **0.004**; shRNA2**: 0.010**; shRNA3: **0.013**; shRNA4: **<0.001**; shRNA5: 0.234 (MWU)	• Unequal variance in ANOVA<br>• shRNA1, unequal variance; shRNA 5, non-normal

Supplemental Table 3. **Overview statistics *in vitro* morphological analysis**. Shown are the statistical analyses (n-number (wells), type of test, Df/F/t-value, *P*-value) for the data shown in Figure 3 and Supplemental Figure 3 (SF3) related to the *in vitro* analysis of *Gpr158* expression and *Gpr158* KD in WT hippocampus primary neurons. Significance (*P*<0.050) is indicated in bold.

**Figure reference**	**n-number**	**type of overall or post-hoc test**	**(Df) & F- or t-value**	***P*-value**	**Remarks**
**4b**: Length (mm)	KO: 10 (3 animals); WT: 10 (4 animals)	ttest	Total: (18) 3.63<br>Apical: (18) 3.76<br>Basal: (18) 0.97	Total: **0.002**<br>Apical: **0.001**<br>Basal: **0.346**	
**4b**: Surface area (mm2)	KO: 10 / 3<br>WT: 10 / 4	ttest	Total: (18) 3.65<br>Apical: (18) 3.73<br>Basal: (18) 1.56	Total: **0.002**<br>Apical: **0.002**<br>Basal: 0.136	
**4b**: Bifurcations (#)	KO: 10 / 3<br>WT: 10 / 4	ttest, MWU	(13.4) 3.76<br>Apical: -<br>Basal: -	Total: **0.002**<br>Apical: **0.001**<br>Basal: 0.481	Total: unequal variance; Apical & basal: non-normal
**4b**: Branches (#)	KO: 10 / 3<br>WT: 10 / 4	ttest, MWU	(14.16) 3.63<br>Apical: -<br>Basal: -	Total: **0.003**<br>Apical: **0.001**, Basal: 0.529	Total: unequal variance; Apical & basal: non-normal
**4c**: AP elicited per current step	KO: 10 / 3<br>WT: 10 / 4	• mixed ANOVA: repeated measure (current injected) ANOVA (genotype)<br>• MWU	• current injection: (15,255) 255.7; genotype x current injected: (15,255) 3.48; genotype: (1,17) 8.12	• current injected: **<0.0001**; current injected x genotype: **<0.0001**; genotype: **0.01;**<br>• 60 pA: **0.03**; 80 pA: **0.02**; 100 pA: **0.01**; 120 pA: **0.02**; 140 pA: **0.045**	• Bonferroni's multiple comparison test
**4d**: Amplitude (mV)	KO: 10 / 3<br>WT: 10 / 4	ttest	(10.7) 0.80	0.432	
**4d**: Threshold (mV)	KO: 10 / 3<br>WT: 10 / 4	ttest	(12.1) -1.06	0.311	
**4d**: Minimal ISI (ms)	KO: 10 / 3<br>WT: 10 / 4	MWU	-	**0.015**	non-normal
**4d**: Rheobase (pA)	KO: 10 / 3<br>WT: 10 / 4	MWU	-	**0.009**	non-normal
**4d**: Input resistance (MOhm)	KO: 10 / 3<br>WT: 10 / 4	ttest	(18) -3.337	**0.004**	
**4d**: Membrane potential (mV)	KO: 10 / 3<br>WT: 10 / 4	MWU	-	**0.035**	non-normal
**S4a**: Minimal ISI @ -70 mV (ms)	KO: 10 / 3<br>WT: 10 / 4	MWU (single-sided)	-	**0.001**	non-normal
**S4b**: Rheobase @ -70 mV (pA)	KO: 9 / 3<br>WT: 10 / 4	t-test (single-sided)	(17) 1.690	**0.055**	

Supplemental Table 4. **Overview statistics *ex vivo* morphological and AP profile analysis**. Shown are the statistical analyses (n-number (cells / animals), type of test, Df/F/t-value, *P*-value) for the data shown in Figure 4 and Supplemental Figure 4, related to the *ex vivo* morphological analysis of *Gpr158* KO and WT neurons in hippocampus CA1 that were analyzed for their intrinsic properties. Significance (*P*<0.050) is indicated in bold.

**Figure reference**	**n-number**	**type of overall or post-hoc test**	**(Df) & F- or t-value**	***P*-value**	**Remarks**
**5a**: Length (mm)	KO: 6<br>WT: 6	• 2-way ANOVA (compartment; genotype)<br>• Bonferroni correction (apical/basal)	• Compartment: (1,23) 219.10; genotype: (1,23) 13.39; interaction: (1,23) 5.22<br>• Apical: (20) 4.204<br>Basal: (20) 0.971	• Compartment: **<0.001**; genotype: **0.002**; interaction: **0.033**<br>• Apical: **0.001**; Basal: 0.686	
**5a**: Surface area (mm2)	KO: 6<br>WT: 6	• 2-way ANOVA (compartment; genotype)<br>• Bonferroni correction (apical/basal)	• Compartment: (1,23) 199.64; genotype: (1,23) 17.73; interaction: (1,23) 4.91<br>• Apical: (20) 4.544<br>Basal: (20) 1.411	• Compartment: **<0.001**; genotype: **0.001**; interaction: **0.001**<br>• Apical: **<0.001**; Basal: 0.347	
**5a**: Bifurcations (#)	KO: 6<br>WT: 6	• 2-way ANOVA (compartment; genotype)<br>• Bonferroni correction (apical/basal)	• Compartment: (1,23) 110.41; genotype: (1,23) 15.00; interaction: (1,23) 3.22<br>• Apical: (20) 4.008<br>Basal: (20) 1.470	• Compartment: **<0.001**; genotype: **0.001**; interaction: <u>0.088</u><br>• Apical: **0.001**; Basal: 0.314	
**5a**: Branches (#)	KO: 6<br>WT: 6	• 2-way ANOVA (compartment; genotype)<br>• Bonferroni correction (apical/basal)	• Compartment: (1,23) 84.43; genotype: (1,23) 14.56; interaction: (1,23) 3.31<br>• Apical: (20) 3.984<br>Basal: (20) 0.347	• Compartment: **<0.001**; genotype: **0.001**; interaction: <u>0.084</u><br>• Apical: **0.002**; Basal: 0.347	

<u>Supplemental Table 5</u>. **Overview statistics of morphological analysis for correlation with behavior**. Shown are the statistical analyses (n-number (animals), type of test, Df/F/t-value, *P*-value (ANOVA; Bonferroni correction) for data shown in Figure 5, related to the *Gpr158* KO and WT mice in which both morphological analysis of CA1 pyramidal neurons and Morris Water maze learning was performed. Significance (*P*<0.050) is indicated in bold, trend (*P*<0.100) in underlined.

Name	symbol	# peptides	CA3 expression	CA1 expression	DG expression	Probe Allen Brain Atlas
Agrin	Agrn	30	high	high	medium	Probe RP_051214_03_B09
Glypican1	Gpc1	10	high	medium	low	Probe RP_051214_03_E04
Glypican2	Gpc2	3	-	-	-	
Glypican3	Gpc3	7	low	low	medium	Probe RP_050329_01_C10
Glypican4	Gpc4	6	-	-	medium	Probe RP_040428_01_C03
Glypican6	Gpc6	8	-	-	-	
Neuropilin1	Nrp1	4	high	low	medium	Probe RP_050915_01_H06
Perlecan	Hspg2	33	-	-	-	
Pikachurin	Egflam	3	-	-	-	
Syndecan4	Sdc4	3	-	-	-	
Spock1/Testican1	Spock1	7	high	low	medium	Probe RP_051101_02_A03
Spock3/Testican3	Spock3	11	high	low	high	Probe RP_050725_01_F03

Supplemental Table 6. **Overview of hippocampus subregion gene expression of ECM-related Gpr158 interactors**. For the published set of 12 ECM-related Gpr158 N-terminal interactors (Catapano and Manji, 2007; Thompson et al., 2008) the level of expression (high; medium; low; absent (-)) in CA3, CA1 and DG is indicated based on specific Allen Brain Atlas probes. Specifically, genes that are expressed in the CA3 region (yellow) are of interest, as they could subserve a similar role in the CA3 to CA1 pathway, as Glypican4 does in the MF to CA3 pathway, as elegantly shown before (Chan et al., 2015). It should be noted however that in total 129 Gpr158 N-terminal interactors were found (Khrimian et al., 2017), increasing the possibility of finding a similar functional pre-postsynaptic pair.

References

Brooks, S. P., Pask, T., Jones, L., and Dunnett, S. B. (2005). Behavioural profiles of inbred mouse strains used as transgenic backgrounds. II: cognitive tests. *Genes Brain Behav.* 4, 307–317. doi:10.1111/j.1601-183X.2004.00109.x.

Condomitti, G., Wierda, K. D., Schroeder, A., Rubio, S. E., Vennekens, K. M., Orlandi, C., et al. (2018). An Input-Specific Orphan Receptor GPR158-HSPG Interaction Organizes Hippocampal Mossy Fiber-CA3 Synapses. *Neuron*. doi:10.1016/j.neuron.2018.08.038.

Khrimian, L., Obri, A., Ramos-Brossier, M., Rousseaud, A., Moriceau, S., Nicot, A.-S., et al. (2017). Gpr158 mediates osteocalcin's regulation of cognition. *J. Exp. Med.*, jem.20171320. doi:10.1084/jem.20171320.

Orlandi, C., Omori, Y., Wang, Y., Cao, Y., Ueno, A., Roux, M. J., et al. (2018). Transsynaptic Binding of Orphan Receptor GPR179 to Dystroglycan-Pikachurin Complex Is Essential for the Synaptic Organization of Photoreceptors. *Cell Rep* 25, 130–145.e5. doi:10.1016/j.celrep.2018.08.068.

Rodgers, R. J., Boullier, E., Chatzimichalaki, P., Cooper, G. D., and Shorten, A. (2002). Contrasting phenotypes of C57BL/6JOlaHsd, 129S2/SvHsd and 129/SvEv mice in two exploration-based tests of anxiety-related behaviour. *Physiol. Behav.* 77, 301–310.

Sutton, L. P., Orlandi, C., Song, C., Oh, W. C., Muntean, B. S., Xie, K., et al. (2018). Orphan receptor GPR158 controls stress-induced depression. *Elife* 7, e33273. doi:10.7554/eLife.33273.

Tarantino, L. M., Gould, T. J., Druhan, J. P., and Bucan, M. (2000). Behavior and mutagenesis screens: the importance of baseline analysis of inbred strains. *Mamm. Genome* 11, 555–564.

Võikar, V., Kõks, S., Vasar, E., and Rauvala, H. (2001). Strain and gender differences in the behavior of mouse lines commonly used in transgenic studies. *Physiol. Behav.* 72, 271–281.

Zeisel, A., Muñoz-Manchado, A. B., Codeluppi, S., Lönnerberg, P., La Manno, G., Juréus, A., et al. (2015). Brain structure. Cell types in the mouse cortex and hippocampus revealed by single-cell RNA-seq. *Science* 347, 1138–1142. doi:10.1126/science.aaa1934
